# GATA-3 in Atlantic salmon (*Salmo salar*): Tissue distribution and its regulation of IL-4/13a promoter

**DOI:** 10.3389/fcimb.2022.1063600

**Published:** 2022-11-14

**Authors:** Heng Chi, Xianghu Meng, Roy Ambli Dalmo

**Affiliations:** ^1^ Laboratory of Pathology and Immunology of Aquatic Animals, KLMME, Ocean University of China, Qingdao, China; ^2^ Norwegian College of Fishery Science, Faculty of Biosciences, Fisheries and Economics, UiT - the Arctic University of Norway, Tromsø, Norway; ^3^ Laboratory for Marine Fisheries Science and Food Production Processes, Qingdao National Laboratory for Marine Science and Technology, Qingdao, China

**Keywords:** GATA-3, IL-4/13a, fish, promoter activity, Th cells, tissue distribution

## Abstract

GATA3 is a transcription factor that plays an important role in T cell lineage differentiation and T-helper 2 (Th2) type immune responses. In this study, we developed two rat antibodies against Atlantic salmon GATA-3 (anti-r*Ss*GATA-3a and anti-r*Ss*GATA-3b, respectively). The western blotting and immunofluorescence results showed that anti-r*Ss*GATA-3b antibodies recognized endogenous *Ss*GATA-3 proteins, while the anti-r*Ss*GATA-3a antibodies did not bind *Ss*GATA-3. Immunohistochemical analysis revealed that *Ss*GATA-3 positive cells were detected in all tissues tested, with relatively high number of immune reactive cells in the gills and spleen. Furthermore, the immunohistochemical study revealed that *Ss*GATA-3 was expressed in pillar cells, epithelial cells, chondrocytes, perichondrium cells, and some undifferentiated basal cells. In addition, we determined 577 bp of the upstream promoter sequence of *Ss*IL-4/13a and found four motifs that matched *Ss*GATA-3 binding sites. The promoter regions of *Ss*IL-4/13a were assessed by transfecting four deletion reporter constructs and *Ss*GATA-3 overexpression plasmids. The result showed that *Ss*GATA-3 enhanced the activity of *Ss*IL-4/13a promoters within the region ranging from -317 to -302 bp upstream of the transcriptional start site. Antibodies against Th2 markers such as GATA-3 are valuable in addressing the diversity of T cell responses in fish.

## Introduction

Naïve T cells, which are functionally immature, can be differentiated into different subsets of effector T cells upon activation in mammals (Zhu and Paul, 2010). CD4^+^ T helper (Th) cells can be subdivided into distinct subsets characterized by specific transcriptional regulatory networks and unique cytokine repertoires, which orchestrate immune responses and play key roles during infection, chronic inflammation, autoimmune diseases, and carcinogenesis (Gagliani and Huber, 2017). The main subsets of T helper cells are Th1/Th2/Th17/Tregs, in which Th1 and Th2 are two well-studied subsets (Zhu J. F. et al., 2012). Th1 cells express T-bet and produce IFN-γ, IL-2, and TNF-α; Th2 cells express GATA-3 and secrete IL-4, IL-5, IL-9, and IL-13. Response governed by Th1 cells is important to mount response against intracellular pathogens such as viruses, bacteria, and microorganisms. Th2 cells are emphasized to be involved in response to extracellular pathogens enabling, e.g., humoral immunity (Kidd, 2003).

GATA-3 is a member of the GATA family of transcription factors and possesses N-terminal transactivation domains and two zinc fingers, namely the N-terminal zinc finger and the C-terminal zinc finger (Fox et al., 1998; Tevosian et al., 1999). The C-terminal region of GATA-3 is highly conserved among the GATA family proteins. The amino acid motif (YxKxHxxxRP) is adjacent to the C-terminal zinc finger domain of GATA-3. It plays a vital role in GATA-3 DNA binding and function, including transcriptional activity and the ability to induce chromatin remodeling at the Th2 cytokine gene loci (Shinnakasu et al., 2006). Several IL-4-like genes such as IL-4/13a, IL-4/13b1, and IL-4/13b2 have been cloned from Atlantic salmon (*Salmo salar* L.), pufferfish (*Tetraodon nigroviridis*), sea bass (*Dicentrarchus labrax* L.), and zebrafish (*Danio rerio*) (Li et al., 2007; Ohtani et al., 2008; Wang et al., 2016; Stocchi et al., 2017). The reports showed that fish IL-4/13a has perfect TATA box and GATA-3 binding motifs in the proximal promoter regions of the gene. However, IL-4/13b lacks such a box and motif in its proximal promoter regions. Thus, IL-4/13a might be the real IL-4 orthologous gene, but more evidence is still needed (Zhu L. Y. et al., 2012).

Cartilaginous and bony fish are the most primitive vertebrates with thymus and possess T-like cells comparable to mammals (Nakanishi et al., 2015). Molecular and cellular evidence for the existence of T-helper cell subsets in fish has appeared during the last years. As examples, the transcription factors (T-bet, GATA-3, Foxp3) and cytokines (IL-12, IFN-γ, IL-4, TGF-β) have all been identified from teleost fish (Neave et al., 1995; Read et al., 2004; Takizawa et al., 2008; Kumari et al., 2009; Wang et al., 2010; Chi et al., 2012), which shows that akin T helper cells differentiation process is also present in fish. Whether these cells will match precisely to the mammalian paradigm and nomenclature remains to be shown, but the findings so far suggest that many similarities to known T cell subsets exist (Wang and Secombes, 2013). This study aimed to investigate the regulatory mechanism of Th2 responses in Atlantic salmon. For this purpose, we prepared rat antibodies of GATA-3 (*Ss*GATA-3) and examined specifically the expression of *Ss*GATA-3 proteins in tissues and the regulation of *Ss*GATA-3 in IL-4/13a (*Ss*IL-4/13a) transcripts.

## Material and methods

### Animals

Atlantic salmon, 40-50g, were kept at the Aquaculture Research Station (Tromsø, Norway) in flat-bottomed circular 200 L tanks at an ambient temperature of approximately 12°C with 12/12 h illumination and fed commercial pelleted diets. Prior to treatment, the fish were anesthetized in Metacaine (50 mg L^-1^, Norsk Medisinaldepot) and sacrificed using 100 mg L^-1^ Metacaine before collecting the different tissues. The production of anti-r*Ss*GATA-3 antibodies in rats were approved by the Instructional Animal Care and Use Committee of the Ocean University of China. All possible endeavors were made to minimize suffering and maintain animal welfare.

### Construction of plasmids

For the construction of the p*Ss*GATA-3-RFP plasmid, the open reading frames of *SsGATA-3* (GenBank No: EU418015) were retrieved from GenBank and cloned as previously described (Kumari et al., 2009). From a spleen cDNA library, the gene was subcloned into a pTagRFP-N vector (Evrogen, Moscow, Russia) by PCR using primer *Ss*GATA3F/*Ss*GATA3R (**Table 1**) to generate p*Ss*GATA-3-RFP plasmids.

**Table 1:** Primers that were used in this study.

Name	Sequence (5’-3’)	Use
*Ss*GATA3F	agctaagcttATGGAAGTATCCGCCGA(HindIII)	Plasmid construction
*Ss*GATA3R	agctaagcttGCCCATGGCAGAGACCA(HindIII)	Plasmid construction
*Ss*GATA3aF	gatatcATGGAAGTATCCGCCGAC(EcoRV)	Plasmid construction
*Ss*GATA3aR	gatatcTCTACCTTCTGAACATGA(EcoRV)	Plasmid construction
*Ss*GATA3bF	gatatcGAGTGCGTTAACTGTGGAG(EcoRV)	Plasmid construction
*Ss*GATA3bR	gatatcGCCCATGGCAGAGACCA(EcoRV)	Plasmid construction
*Ss*IL-4/13aproF	TCCTACCTGCACTGAGTGTCGGA	5’-flanking regions cloning
*Ss*IL-4/13aproR	CATCTTTGGTTGGGTTTATTTG	5’-flanking regions cloning
*Ss*IL-4/13aF1	agctaagcttTCCTACCTGCACTGAGT(HindIII)	Promoter cloning
*Ss*IL-4/13aF2	agctaagcttATTGGCAGATAAGACCT(HindIII)	Promoter cloning
*Ss*IL-4/13aF3	agctaagcttCTTGAGGCCCCGTCGTT(HindIII)	Promoter cloning
*Ss*IL-4/13aF4	agctaagcttATTCGGCGAAACGCCTCT(HindIII)	Promoter cloning
*Ss*IL-4/13aR	cgggcccgcgCATCTTTGGTTGGGTTTA(SacII)	Promoter cloning

The endonucleases are marked at the end of the sequence and restriction sites are shown in lower cases.

For the construction of pET*Ss*GATA-3a and pET*Ss*GATA-3b, which expresses His-tagged recombinant *Ss*GATA-3a (r*Ss*GATA-3a, amino acid residues 1-260) and *Ss*GATA-3b (r*Ss*GATA-3b, amino acid residues 261-441), the coding sequences of *SsGATA-3a* and *SsGATA-3b* were amplified by PCR with primers *Ss*GATA3aF/*Ss*GATA3aR and *Ss*GATA3bF/*Ss*GATA3bR (**Table 1**). The PCR products were ligated with the TA cloning vector T-Simple (TransGen, Beijing, China). The recombinant plasmids were digested with EcoRV to retrieve fragments containing *SsGATA-3a* and *SsGATA-3b*, which were inserted into pET259 at the EcoRV site to obtain pET*Ss*GATA-3a and pET*Ss*GATA-3b.

For the construction of *Ss*IL-4/13a reporter gene plasmids, the 5’-flanking region sequence of the *SsIL-4/13a* (GenBank No: NM_001204895.1) was obtained from Genome (GenBank NO: NC_059454.1) according to the BLASTN programs. The Genomic DNA was isolated from spleen with the TIANNamp Marine Animals DNA kit (TransGen, Beijing, China). PCR amplified approximately 600 bp of 5’-flanking regions by primer *Ss*IL-4/13aproF/*Ss*IL-4/13aproR (**Table 1**). For sequencing, the PCR products were cloned in TA cloning vector T-Simple (TransGen, Beijing, China). The Identification of transcription factor binding motifs was predicted with TRANSFAC^®^ (Biobase International) (Heinemeyer et al., 1998) and MatInspector version 6.2 (Cartharius et al., 2005). Sequences with successive removal of the 5’-flanking regions were amplified by PCR using forward primers *Ss*IL-4/13aF1, *Ss*IL-4/13aF2, *Ss*IL-4/13aF3, *Ss*IL-4/13aF4 and reverse primer *Ss*IL-4/13aR (**Table 1**) to generate deletion constructs p(-577/+20) Luc, p(-317/+20) Luc, p(-302/+20) Luc and p(-264/+20) Luc. The PCR products were inserted into reporter vector pMetLuc-2 (Clontech, Mountain View, CA, USA). All plasmids were transformed and grown in One Shot TOPO 10 *Escherichia coli* (Invitrogen, Carlsbad, CA, USA) and then isolated using Endo-free Plasmid Mini Kit (QIAgen, Hilden, Germany), verified by restriction map analysis and DNA sequencing.

### Preparation of recombinant proteins and antibodies

The r*Ss*GATA-3a and r*Ss*GATA-3b were expressed and purified as described previously (Wang and Sun, 2016). Briefly, *Escherichia coli* BL21 (DE3) cells (TransGen, Beijing, China) were transformed separately with pET*Ss*GATA-3a and pET*Ss*GATA-3b. Then, the transformants were grown to the mid-logarithmic phase in LB medium at 37 °C, and isopropyl-β-dithiogalactopyranoside was added to the cultures to a final concentration of 1 mM. After growing at 30°C for an additional 6 hours, the cells were harvested by centrifugation (3000 g). His-tagged proteins were purified using Ni-NTA Agarose (QIAgen, Hilden, Germany) as recommended by the manufacturer. The proteins were concentrated with PEG20000 (Solarbio, Beijing, China), and endotoxins were removed as reported previously (Zhang and Sun, 2019). The concentrated proteins were analyzed by sodium dodecyl sulfate-polyacrylamide gel electrophoresis (SDS-PAGE), and their concentrations were determined using the Bradford method with bovine serum albumin as a standard (Bradford, 1976).

The rat anti-r*Ss*GATA-3a and rat anti-r*Ss*GATA-3b serum were prepared following a protocol described previously (Liu et al., 2010). The antibodies were purified using Protein G-based Chromatography Media (Sigma, St. Louis, MO, USA) in line with the introduction of manufacture. The concentrations of purified antibodies were determined using the Bradford method with bovine serum albumin as a standard and adjusted to 1 mg ml^-1^ (Bradford, 1976). The specificity of antibodies was detected by Western blotting and immunofluorescence.

### Western blotting

Tissue and recombinant proteins were electro-transformed (30 V for 1.5 h) from a 12.5% SDS-PAGE onto a polyvinylidene fluoride (PVDF) membrane. Then the membrane was blocked with 5% skimmed milk for 1 hour at 37°C and incubated with rat anti-r*Ss*GATA-3a (1:1000) or rat anti-r*Ss*GATA-3b (1:1000) overnight at 4°C, respectively. After being washed thrice with PBST, the membrane was incubated with goat-anti-rat Ig-HRP (1:5000) (Santa Cruz Biotechnology, Santa Cruz, CA, USA) for 1 hour at 37°C. After washing three times with PBST, the immunocomplex was detected using Pierce™ ECL Western Blotting Substrate (Pierce, Rockford, IL, USA).

### Cell line culture, transfection, immunofluorescence, and reporter activity assay

Chinook salmon embryonic (CHSE-214) cells were seeded in the standard flask (Nunc™) with L-15 medium (Invitrogen, Carlsbad, CA, USA) containing penicillin (60 μg ml^−1^), streptomycin (100 μg ml^−1^), 1% non-essential amino acids and 8% fetal bovine serum (FBS). The CHSE-214 cells were incubated at 20°C for one week. Cells were washed twice with 10 ml PBS before adding 1.5 ml trypsin (Sigma, St. Louis, MO, USA). Loosened cells were re-suspended in L-15 medium (8% FBS, 1% NEAA, without antibiotics), counted in Nucleocounter YC-100 (Invitrogen, Carlsbad, CA, USA), and seeded in separate wells (1 x 10^5^ cells well^-1^). The p*Ss*GATA-3-RFP and pTagRFP-N were transfected to cells with Lipofectamine LTX (Invitrogen, Carlsbad, CA, USA) according to supplier protocol. Mock-transfected cells were considered as control. After 48 h, the cells were fixed with 4% formaldehyde (w/v) (Invitrogen, Carlsbad, CA, USA) for 30 minutes. After washing thrice, the cells were incubated with 200 μl 5% skimmed milk for 2 h at room temperature. The cells were subsequently incubated with 200 μl rat anti-r*Ss*GATA-3a (1:200), rat anti-r*Ss*GATA-3b (1:200) or rat anti-Trx (1:200) as control for 1 h at 37°C with occasional shaking, then washed thrice with PBS and incubated for another 1 h at 37°C with goat-anti-rat Ig-FITC (1:500) (Abcam, Boston, MA, USA). After washing thrice, DAPI (Sigma, St. Louis, MO, USA) was used for nucleic acid staining. Micrographs were obtained by an inverted fluorescence microscope equipped with DAPI-365 and Texas Red 530-585 filters (Carl Zeiss Imager A2, Jena, Germany).

For reporter activity assay, the CHSE-214 cells were re-suspended in L-15 medium and seeded in 24 well culture plate (2 x 10^5^ cells well^-1^). The reporter vectors, p*Ss*GATA-3 and pTagRFP**-**N were transfected to the cells with Lipofectamine LTX according to the manufacturer’s instructions. 48 hours after transfection, the culture mediums of transfected cells were analyzed for luciferase activity and SEAP activity using the luciferase assay kit and great scape ™ SEAP chemiluminescent detection kit (Clontech, Mountain View, CA, USA), respectively.

### Immunohistochemistry

Four-µm-thick formalin-fixed, paraffin-embedded (FFPE) tissue sections were mounted on StarFrost Advanced Adhesive slides (Engelbrecht, Kassel, Germany), followed by drying at 60°C for 2 h. After deparaffinization, slides were pretreated at 120°C in Citric acid buffer (pH=6.0) for 10 min to retrieve antigens. Sections were incubated for 20 min at room temperature in a humid chamber with 1% H_2_O_2_ and 100% methanol to quench endogenous peroxidase. After washing thrice, the sections were incubated with 5% skimmed milk for 2 h at room temperature. Then the sections were incubated with rat anti-r*Ss*GATA-3b (1:200) or rat anti-Trx (1:200) overnight at 4°C, then washed thrice with PBS, and incubated for another 1 h at 37°C with goat-anti-rat Ig-HRP (1:500) (Abcam, Eugene, USA). After washing thrice, the tissue sections were immersed in a freshly prepared 3, 3’-diaminobenzidine (DAB) substrate reagent solution (Invitrogen, Carlsbad, CA, USA) for 10 minutes. Micrographs were obtained by microscope (Carl Zeiss Imager A2, Jena, Germany).

### Statistical analysis

Statistical analysis was performed using one-way ANOVA and LSD multiple comparisons in SPSS 20.0 software (SPSS Inc., Chicago, IL, USA). Data are expressed as mean ± SD, and statistical significance was defined as *P* < 0.01.

## Results

### Expression of r*Ss*GATA-3a and r*Ss*GATA-3b protein and detection of the specificity of the antibodies

**Figure 1:**
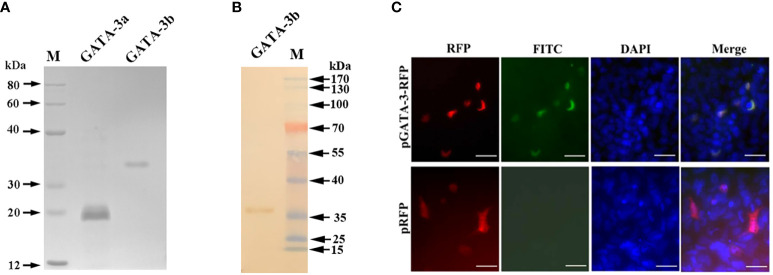
Production and validation of anti-r*Ss*GATA-3 antibodies. **(A)** The purified r*Ss*GATA-3 proteins were shown by SDS-Page. Lane M: marker; Lane GATA-3a: purified r*Ss*GATA-3a proteins; Lane GATA-3b: purified r*Ss*GATA-3b proteins. **(B)** Western blotting shows the specificity of the rat anti-r*Ss*GATA-3b antibodies. Lane M: marker; Lane GATA-3b: r*Ss*GATA-3b proteins incubated with purified anti-r*Ss*GATA-3b antibodies. **(C)** Overexpression of *Ss*GATA-3-RFP in CHSE-214 cells for detection of the specificity of anti-r*Ss*GATA-3b antibodies. The nuclei were stained with DAPI. Bar = 10 μm.

The r*Ss*GATA-3a and r*Ss*GATA-3b proteins were expressed in and purified from transfected *E. coli* as His-tagged proteins, respectively (**Figure 1A**), and the anti-r*Ss*GATA-3a and anti-r*Ss*GATA-3b antibodies were prepared from rat. Western blotting showed that the rat anti-r*Ss*GATA-3b antibodies could specifically recognize r*Ss*GATA-3b (**Figure 1B**). To further examine the specificity of the rat anti-r*Ss*GATA-3b antibodies, CHSE-214 cells were transfected with p*Ss*GATA-3-RFP or pTagRFP-N. Fluorescence microscopy results revealed that *Ss*GATA-3-RFP (red) in cells with p*Ss*GATA-3-RFP transfection were identified in or close to the nuclei (blue) and could be specifically detected by rat anti-r*Ss*GATA-3b antibodies (green) (**Figure 1C**). In contrast, red fluorescence protein in the pTagRFP-N transfection group was uniformly expressed in cells, but no green color was observed (**Figure 1C**). However, the anti-*Ss*GATA-3a antibodies did not seem to bind GATA-3 (result not shown).

### Localization of *Ss*GATA-3 proteins in Atlantic salmon tissues

**Figure 2:**
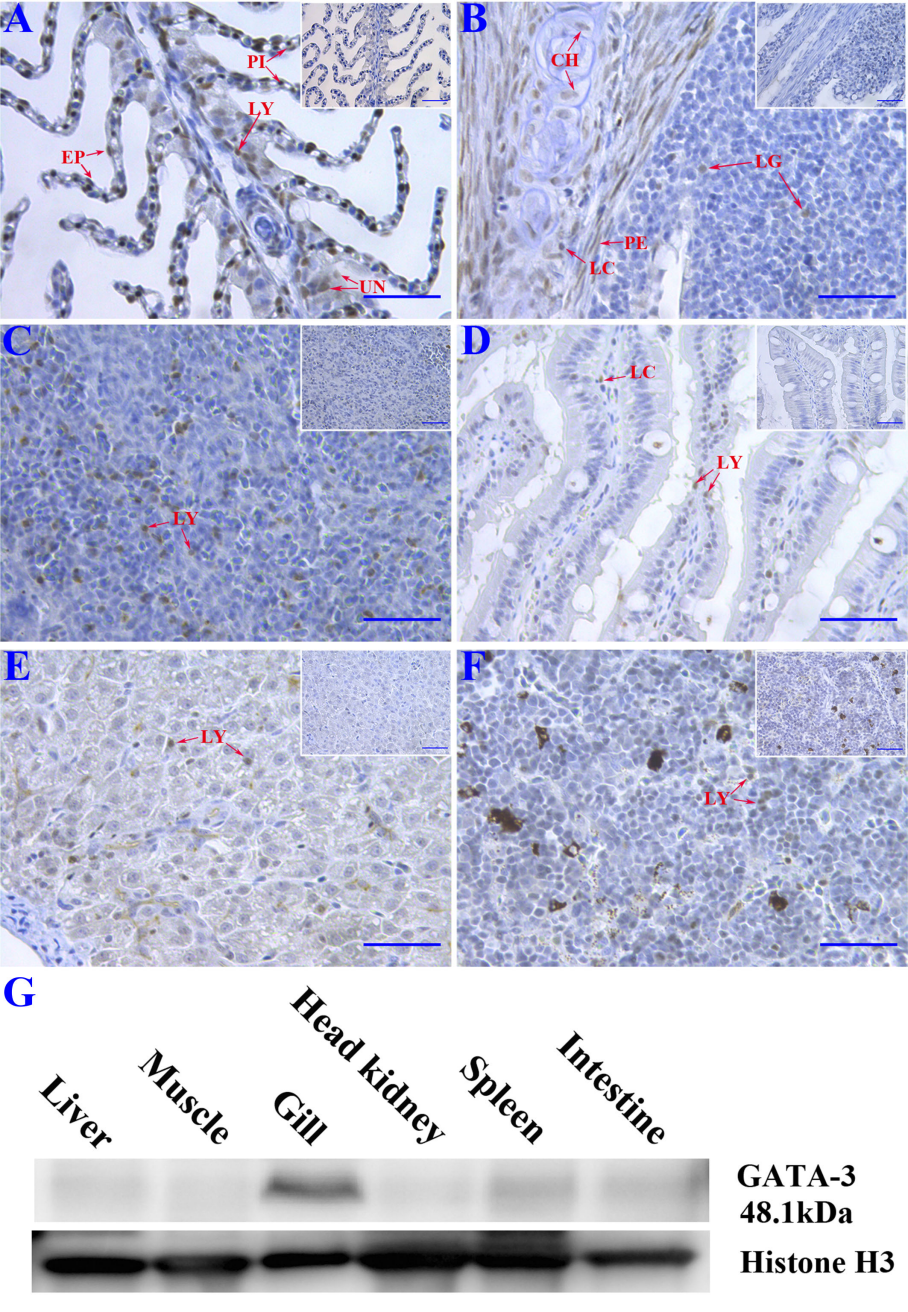
Immunohistochemistry study of the distribution of SsGATA-3 proteins in gills **(A)**, gill-associated lymphoid tissue (GIALT) **(B)**, spleen **(C)**, intestine **(D)**, liver **(E)**, and head kidney **(F)**. PI: pillar cell; LY: Lymphocyte-like cell; EP: Epithelial cell; UN: undifferentiated basal cell; CH: chondrocyte; LC: Lymphocyte-like cell in the capillary vessel; PE: perichondrial cell; LG: Lymphocyte-like cell in GIALT. Micrographs displayed in the box show negative controls. Bar=50μm. **(G)** Western blot showing the distribution of endogenous SsGATA-3 in different tissues. Anti-histone 3 was used for loading control.

Immunohistochemistry and Western blotting were conducted to detect tissue distribution of *Ss*GATA-3 in healthy Atlantic salmon tissues. The gray color corresponding to the immunocomplex was observed in the gills, gill associated lymphoid tissue (GIALT), intestine, spleen, liver, and head kidney (**Figures 2A-F**). In gills and GIALT, the *Ss*GATA-3 proteins were expressed in pillar cells, lymphocyte-like cells, epithelial cells, chondrocytes, perichondrial cells, and some undifferentiated basal cells. In the spleen, intestine, liver, and head kidney, the SsGATA-3 positive cells were mainly found in lymphocyte-like cells. The results of Western blot as shown in **Figure 2G**, the *Ss*GATA-3 protein was found in most of the analyzed tissues. The highest contents of *Ss*GATA-3 proteins were revealed in the gills, followed by spleen, intestine, liver, head kidney, and liver in healthy salmon. The level of *Ss*GATA-3 protein in muscle was low.

### Effect of *Ss*GATA-3 proteins on the promoter activity of *Ss*IL-4/13a cytokine

**Figure 3:**
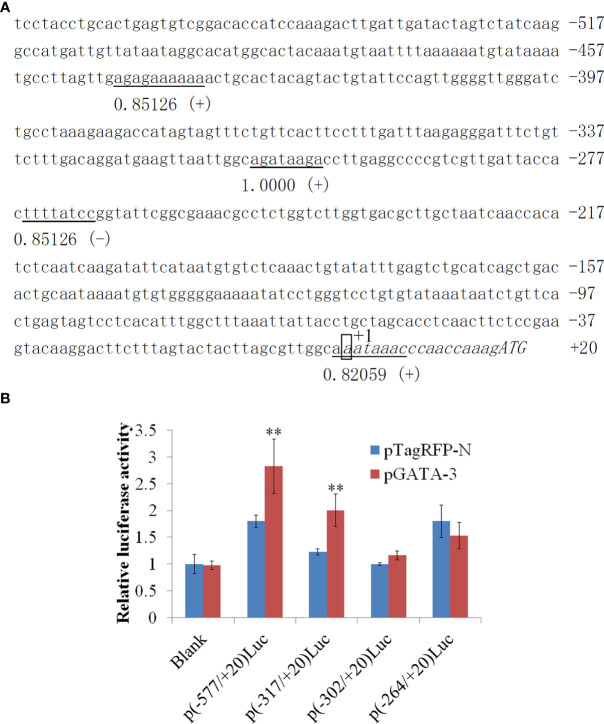
The sequence and activity of salmon IL-4/13a promoter. **(A)** The nucleotide sequence of promoter regions (557 bp) was obtained. The transcription start site is designated as +1 and boxed. Transcription factor binding sites were predicted by MatInspector and TRANSFAC^®^. Consensus elements of transcription factor binding sites are underlined. The number under horizontal lines indicates matrix similarity. The symbol (+) indicates the binding sites identified on the positive strand, whereas the symbol (-) indicates the opposite. **(B)** CHSE-214 cells were transfected by pLuc*Ss*IL-4/13a reporter vectors with p*Ss*GATA-3 or pTagRFP-N. pSeap-Control performed as a normalized control. Luciferase activity is expressed relative to SEAP (mean ± SD from six wells). Double asterisks (**) above bars indicate significant differences (*P* < 0.01). Data are from six wells of cells per treatment in one experiment and represent three independent experiments.

In 577 bp 5’-flanking regions (5’-FRs) of *Ss*IL-4/13a, which exhibit four GATA-3 binding sites located in -445/-339, -310/-303, -268/-261, and -2/+7 (**Figure 3A**). In this study, we examined the potential effect of *Ss*GATA-3 on the activity of the *Ss*IL-4/13a promoter. For this purpose, four promoter reporter plasmids contain 577bp, 317bp, 302 bp or 264 bp 5’-FRs of *Ss*IL-4/13a were created, in which the promoter activities were reflected by the activities of the luciferase reporter. CHSE-214 cells were transfected with p*Ss*GATA-3 plus four pLuc*Ss*IL-4/13a reporter vectors respectively, and the luciferase activities were determined. The results showed that luciferase activities were significantly increased in the presence of p*Ss*GATA-3 compared to the cells with pTagRFP-N p (-577/+20) luc and p (-317/+20) luc transfectants (**Figure 3B**). However, in p (-302/+20) luc and p (-264/+20) luc transfectants, luciferase activities were not significantly different between the groups in the presence of p*Ss*GATA-3 and pTagRFP-N (**Figure 3B**).

## Discussion

GATA-3 is a crucial regulator of both innate and adaptive immunity, and has an important role in the development and function of T cells, B cells, and nonhematopoietic cells (central nervous system, inner ear, and skin tissues) in embryonic and adult tissues (Wan, 2014). In this report, we prepared two fragments of *Ss*GATA-3 recombinant proteins (r*Ss*GATA-3a and r*Ss*GATA-3b) and obtained their specific rat antibodies (rat anti-r*Ss*GATA-3a and anti-r*Ss*GATA-3b). The rat anti-r*Ss*GATA-3b antibodies bound specifically to *Ss*GATA-3.

The tissue distribution of *Ss*GATA-3 proteins was comparable with the expression pattern of *SsGATA-3* mRNA in various tissues, where *SsGATA-3* previously has been shown to be highly expressed in head kidney, spleen, and gill (Kumari et al., 2009). Head kidney and spleen are major immune organs that produce various immune-related cytokines in teleost fish (Zapata et al., 2006; Ohtani et al., 2008; Zhu L. Y. et al., 2012). Gills and GIALT are essential components of mucosal immunity in fish, with different types of immune cells present (Liang et al., 2022). Such lymphoid aggregations are important sites for immune cell development, settlement, proliferation, and the generation of immune responses. Gill and GIALT with high expression of *GATA-3* mRNA (Kumari et al., 2009) and large number of GATA-3 positive cells shown in the current study may be an indication that GATA-3 is likely associated with the mucosal immune response of teleost fish.

In mammals, Th2 immune response is characterized by Th2 cells, eosinophils, mast cells, and basophils. IL-4, IL-5, IL-9, and IL-13 activate B cells and macrophages, which can either be protective or have adverse effects in immune response (Wynn, 2015). Our previous study showed that overexpression of GATA-3 *in vivo* upregulated *IL-4/13a* expression in salmon (Slettjord et al., 2020). In this study, we found that GATA-3 increased the promoter activity of *IL-4/13a in vitro* and the binding site was between -317 and -302 bp upstream of the transcriptional start site. This finding suggests that Th2 immune response may exist in fish where GATA-3 plays an important role. Of course, the expression of GATA-3 and its related Th2 regulatory molecular pathways in fish still need to be further studied, such as whether it is regulated by non-coding RNAs, such as miRNA and lncRNA (Zhou et al., 2018; Zhang et al., 2020), and whether it is related to cellular processes such as cell proliferation, apoptosis, and autophagy (Li et al., 2021; Zhou et al., 2022).

In summary, *Ss*GATA-3 protein may likely regulate Th2 immune response by modulating the promoter activity of *Ss*IL-4/13a cytokine in salmon. The *Ss*GATA-3 positive cells were mainly present in lymphocyte-like cells in spleen, intestine, liver, and head kidney. However, the *Ss*GATA-3 proteins were also expressed in pillar cells, lymphocytes, epithelial cells, chondrocytes, perichondrium cells and some undifferentiated basal cells. This result indicated that not only does GATA-3 participate in common lymphoid progenitor to Th2, the activity of GATA-3 may also extend beyond a CD4^+^ T response.

## Data availability statement

The original contributions presented in the study are included in the article/supplementary material. Further inquiries can be directed to the corresponding authors.

## Ethics statement

The animal study was reviewed and approved by the Instructional Animal Care and Use Committee of the Ocean University of China. The experimental protocols used in this study for Atlantic salmon were beforehand approved by the Norwegian Food Safety Authority.

## Author contributions

HC and RD participated in the conception and design of this study; HC and XM performed the experiments, analyzed the experimental data, and wrote the original draft; RD reviewed and edited the manuscript; All authors read and approved this version of the final manuscript.

## Funding

This research was jointly supported by the grants from the National Key Research and Development Program of China (2019YFD0900101) and the National Natural Science Foundation of China (31872594, 41906108, and 31730101). The Research Council of Norway provided financial support to the study through project no. 239140.

## Conflict of interest

The authors declare that the research was conducted in the absence of any commercial or financial relationships that could be construed as a potential conflict of interest.

## Publisher’s note

All claims expressed in this article are solely those of the authors and do not necessarily represent those of their affiliated organizations, or those of the publisher, the editors and the reviewers. Any product that may be evaluated in this article, or claim that may be made by its manufacturer, is not guaranteed or endorsed by the publisher.
